# Photobiomodulation Therapy Reduces Oxidative Stress and Inflammation to Alleviate the Cardiotoxic Effects of Doxorubicin in Human Stem Cell-Derived Ventricular Cardiomyocytes

**DOI:** 10.3390/biomedicines13071781

**Published:** 2025-07-21

**Authors:** Guilherme Rabelo Nasuk, Leonardo Paroche de Matos, Allan Luís Barboza Atum, Bruna Calixto de Jesus, Julio Gustavo Cardoso Batista, Gabriel Almeida da Silva, Antonio Henrique Martins, Maria Laura Alchorne Trivelin, Cinthya Cosme Gutierrez Duran, Ana Paula Ligeiro de Oliveira, Renato de Araújo Prates, Rodrigo Labat Marcos, Stella Regina Zamuner, Ovidiu Constantin Baltatu, José Antônio Silva

**Affiliations:** 1 Biophotonics-Medicine Postgraduate Program, Universidade Nove de Julho (UNINOVE), São Paulo 01504-001, Brazil; guilhermenike@uninove.edu.br (G.R.N.); leonardoparoche@gmail.com (L.P.d.M.); b.calixto@uni9.edu.br (B.C.d.J.); julio.batista@uni9.edu.br (J.G.C.B.); almeidagabriel06@uni9.edu.br (G.A.d.S.); maria.alchorne.trivelin@uni9.pro.br (M.L.A.T.); cduran@uni9.pro.br (C.C.G.D.); apligeiro@uni9.pro.br (A.P.L.d.O.); pratesra@uni9.pro.br (R.d.A.P.); labat@uni9.pro.br (R.L.M.); stella.rz@uni9.pro.br (S.R.Z.); 2 Medicine Postgraduate Program, Universidade Nove de Julho (UNINOVE), São Paulo 01504-001, Brazil; allan.atum@uni9.pro.br; 3 Department of Pharmacology and Toxicology, University of Puerto Rico, Mayaguez, PR 00681, USA; antonio.martins@upr.edu; 4 Center of Innovation, Technology and Education (CITE), Anhembi Morumbi University, Anima Institute, Sao Jose dos Campos Technology Park, Sao Jose dos Campos 12247-016, Brazil; obaltatu@alfaisal.edu; 5 College of Medicine, Alfaisal University, Riyadh 11533, Saudi Arabia

**Keywords:** photobiomodulation, cardiomyocytes, doxorubicin, oxidative stress, cardiotoxicity

## Abstract

**Background/Objectives:** Doxorubicin (DOX), a widely used anthracycline chemotherapeutic agent, is recognized for its efficacy in treating various malignancies. However, its clinical application is critically limited due to dose-dependent cardiotoxicity, predominantly induced by oxidative stress and compromised antioxidant defenses. Photobiomodulation (PBM), a non-invasive intervention that utilizes low-intensity light, has emerged as a promising therapeutic modality in regenerative medicine, demonstrating benefits such as enhanced tissue repair, reduced inflammation, and protection against oxidative damage. This investigation sought to evaluate the cardioprotective effects of PBM preconditioning in human-induced pluripotent stem cell-derived ventricular cardiomyocytes (hiPSC-vCMs) subjected to DOX-induced toxicity. **Methods:** Human iPSC-vCMs were allocated into three experimental groups: control cells (untreated), DOX-treated cells (exposed to 2 μM DOX for 24 h), and PBM+DOX-treated cells (preconditioned with PBM, utilizing 660 nm ±10 nm LED light at an intensity of 10 mW/cm^2^ for 500 s, delivering an energy dose of 5 J/cm^2^, followed by DOX exposure). Cell viability assessments were conducted in conjunction with evaluations of oxidative stress markers, including antioxidant enzyme activities and malondialdehyde (MDA) levels. Furthermore, transcriptional profiling of 40 genes implicated in cardiac dysfunction was performed using TaqMan quantitative polymerase chain reaction (qPCR), complemented by analyses of protein expression for markers of cardiac stress, inflammation, and apoptosis. **Results:** Exposure to DOX markedly reduced the viability of hiPSC-vCMs. The cells exhibited significant alterations in the expression of 32 out of 40 genes (80%) after DOX exposure, reflecting the upregulation of markers associated with apoptosis, inflammation, and adverse cardiac remodeling. PBM preconditioning partially restored the cell viability, modulating the expression of 20 genes (50%), effectively counteracting a substantial proportion of the dysregulation induced by DOX. Notably, PBM enhanced the expression of genes responsible for antioxidant defense, augmented antioxidant enzyme activity, and reduced oxidative stress indicators such as MDA levels. Additional benefits included downregulating stress-related mRNA markers (*HSP1A1* and *TNC*) and apoptotic markers (*BAX* and *TP53*). PBM also demonstrated gene reprogramming effects in ventricular cells, encompassing regulatory changes in *NPPA, NPPB,* and *MYH6*. PBM reduced the protein expression levels of IL-6, TNF, and apoptotic markers in alignment with their corresponding mRNA expression profiles. Notably, PBM preconditioning showed a diminished expression of BNP, emphasizing its positive impact on mitigating cardiac stress. **Conclusions:** This study demonstrates that PBM preconditioning is an effective strategy for reducing DOX-induced chemotherapy-related cardiotoxicity by enhancing cell viability and modulating signaling pathways associated with oxidative stress, as well as inflammatory and hypertrophic markers.

## 1. Introduction

Doxorubicin {DOX, (7S, 9S)-7-[((2S, 4S, 5S, 6S)-4-amino-5-hydroxy-6-methyloxan-2-yl]oxy-6,9,11-trihydroxy-9-(2-hydroxyethyl)-4-methoxy-8,10-dihydro-7H-tetraceno-5,12-dione}, a potent anthracycline chemotherapeutic agent, effectively targets multiple cancers, including breast and bladder cancer, Kaposi’s sarcoma, lymphoma, and lymphocytic leukemia (for a review, see [1]). Due to its well-established efficacy, it is included in the World Health Organization’s (WHO’s) list of essential medicines. Although the WHO acknowledges the safety concerns associated with its use, the clinical use of DOX remains critically constrained by its dose-dependent cardiotoxicity, which can lead to heart damage such as cardiomyopathy and heart failure [2]. As cancer survival rates steadily improve, the long-term risks associated with anthracycline treatment have drawn increasing attention, emphasizing the urgent need for innovative therapeutic strategies that mitigate chemotherapy-induced cardiac damage while preserving the efficacy of cancer treatment [3].

Acute cardiotoxicity related to DOX usually occurs during treatment or shortly afterward and is marked by vasodilation, low blood pressure, and temporary impairments [4]. Acute toxicity can optimally be managed pharmacologically using dexrazoxane and other agents [5,6]. Early disease diagnosis is essential to enable treatment during this reversible phase. Cardiac surveillance should not exclusively rely on symptoms, as this approach may lead to missed opportunities for early intervention [6]. Chronic cardiotoxicity, which can manifest during treatment or within weeks to months thereafter, encompasses conditions such as dilated cardiomyopathy, reduced ventricular function, and congestive heart failure and may ultimately progress to mortality. Detecting preclinical cardiotoxicity is a key goal in anthracycline therapy, presenting significant long-term therapeutic challenges [7,8,9].

Human pluripotent stem cells (hPSCs) have been investigated for potential use in repairing heart tissue affected by aging or disease. Induced pluripotent stem cells (iPSCs) can accurately model cardiovascular diseases, supporting personalized research into these conditions [10]. Cardiomyocytes derived from hPSCs have demonstrated the ability to restore function in injured heart tissue, underscoring their therapeutic potential [11].

Photobiomodulation (PBM) is a therapy that utilizes low-level laser or LED light to stimulate biological processes, representing a significant advancement in regenerative medicine [12,13]. The process involves molecules such as cytochrome c oxidase in the mitochondrial respiratory chain absorbing red and near-infrared (NIR) light. This enhances adenosine triphosphate (ATP) production, resulting in effects such as calcium release and alterations in gene expression in different diseases, including complex conditions such as Alzheimer’s disease [14]. These effects enhance tissue repair, lower inflammation, and defend against oxidative stress [15,16].

Building on previous findings that PBM therapy enhances nitric oxide (NO) production and improves the survival of human-induced pluripotent stem cell-derived ventricular cardiomyocytes (hiPSC-vCMs) under doxorubicin-induced oxidative stress [12], this study investigates the broader potential of PBM preconditioning. The DOX concentration derived from neonatal rat ventricular myocytes, immortalized cardiomyoblasts, and cardiomyocyte-derived cell lines is within physiological steady-state levels (0.5–2 μM) [17]. In comparison, the peak plasma concentration in humans is approximately 5 μM [18].

This study aims to investigate the efficacy of PBM preconditioning in mitigating DOX-induced cardiotoxicity by examining transcriptional alterations and stress markers associated with cardiac dysfunction signaling.

## 2. Materials and Methods

### 2.1. Study Sample

This study utilized human ventricular cardiomyocytes (hiPSC-vCMs) derived from induced pluripotent stem cells, which were subjected to photobiomodulation (PBM) treatment followed by exposure to doxorubicin (DOX). The hiPSC-vCMs were sourced from PluriCardio at PluriCell Biotech, São Paulo, Brazil. The cells were thawed and cultured according to the supplier’s protocol and then plated on Geltrex-coated plates (Thermo Fisher Scientific, Waltham, MA, USA) at a density of 2.5 × 10^5^ cells per well in a 6-well microplate, using 1 mL of cardiomyocyte plating medium. Forty-eight hours post-seeding, the medium was replaced with cardiomyocyte maintenance medium, and the experiments started 24 h later. The cells were cultured in a controlled environment set at 37 °C with 5% CO_2_ to maintain optimal growth conditions. The experimental setup comprised three distinct groups: a control group with untreated cells, a group treated with 2 μM doxorubicin (DOX) for 24 h, and a group preconditioned with photobiomodulation (PBM) before DOX exposure to evaluate its potential protective benefits. Each experiment was conducted in triplicate to ensure the accuracy and reproducibility of the results.

### 2.2. Photobiomodulation (PBM) Protocol

PBM was performed immediately before DOX exposure using an LED plate (Bio Lambda, São Paulo, Brazil) emitting red light at 660 nm ± 10 nm, with an intensity of 10 mW/cm^2^. Each well was irradiated for 500 s, delivering a total energy dose of 5 J/cm^2^. The light source irradiated the entire well surface from a constant distance of 14 mm, producing a beam spot size of 1.13 cm^2^ at the culture surface and covering an area of 0.78 cm^2^. Calibration of the LED device was performed by the manufacturer immediately before this study. Irradiation was conducted in a dark environment to eliminate light interference.

### 2.3. Doxorubicin Exposure

For DOX treatment, cells in the DOX and PBM+DOX groups were exposed to 2 μM doxorubicin in a cardiomyocyte maintenance medium. The control cells were treated with an equal volume of sterile saline. After 24 h, the supernatants were centrifuged at 500× *g* for 10 min at 4 °C. The cells were then frozen in liquid nitrogen and stored at −80 °C for further analysis.

### 2.4. Cell Viability Assessment

An MTT (3-(4,5-dimethylthiazol-2-yl)-2,5-diphenyltetrazolium bromide) assay (Sigma-Aldrich, St. Louis, MO, USA) was employed to evaluate cell viability. The cells were plated at a density of 1 × 10^4^ and incubated for 24 h at 37 °C in a humidified incubator with 5% CO_2_. After incubation, 20 μL of 5 mg/mL MTT solution was added to each well and incubated for 4 h. Post-incubation, the supernatant was removed, and dimethyl sulfoxide (DMSO) was added to dissolve the insoluble formazan product. Absorbance was measured at 570 nm using a Varioskan spectrophotometer (Thermo Fisher Scientific, Waltham, MA, USA). A cell viability analysis was conducted in triplicate across three different experimental protocols.

### 2.5. Quantitative PCR for Gene Expression

#### 2.5.1. RNA Extraction and Reverse Transcription

Total RNA was extracted using Trizol^®^ Reagent (Invitrogen, Thermo Fisher Scientific, Waltham, MA, USA). RNA was precipitated with isopropanol, washed with ethanol, and resuspended in diethylpyrocarbonate-treated water. RNA purity and concentration were confirmed using a NanoDrop ND-2000 spectrophotometer. Genomic DNA was eliminated via DNase I treatment. Complementary DNA (cDNA) was synthesized and stored at −20 °C for further analysis.

#### 2.5.2. Quantitative Polymerase Chain Reaction (PCR)–qPCR

Gene expression was analyzed using quantitative PCR (qPCR) with Custom TaqMan Array Plates (Thermo Fisher Scientific, Carlsbad, CA, USA), which contained 40 genes related to the cardiac signaling pathway. The qPCR reactions employed TaqMan Universal Fast Master Mix 2X Solution, nuclease-free water, and 1 µg of cDNA per well. Reactions were conducted in an ABI Prism 7500 Fast system (Applied Biosystems, Thermo Fisher, Waltham, MA, USA) under the following conditions: initial denaturation at 95 °C for 20 s, followed by 40 cycles at 95 °C for 3 s and 60 °C for 30 s. ROX dye was incorporated in all reactions to account for volume discrepancies and evaporation. The quantification cycle (Cq) was documented as the cycle number at which the fluorescent signal surpasses the detection threshold. For reference gene selection, both *18S* rRNA and *GAPDH* were evaluated. *18S* rRNA demonstrated stable expression across all experimental conditions (coefficient of variation < 5%), while *GAPDH* showed higher variability (coefficient of variation > 15%). Therefore, *18S* rRNA was selected as the reference gene for normalization, and *GAPDH* was analyzed solely as a metabolic pathway gene. Target gene expression was normalized by calculating ΔCq values (target gene Cq minus average *18S* rRNA Cq), and relative gene expression was determined using the 2^−ΔΔCq^ method and reported as fold changes. All qPCR assays were performed in duplicate and repeated three times per sample. Data analysis was executed using SDS 1.4 Software (Applied Biosystems, Thermo Fisher, Waltham, MA, USA). Quantitative polymerase chain reaction (qPCR) assays were conducted in duplicate and repeated three times for each sample.

### 2.6. Analysis of Oxidative Stress-Related Enzymes

Oxidative stress-related enzymes, including superoxide dismutase (SOD), malondialdehyde (MDA), and glutathione peroxidase (GPx), were assessed. SOD activity was measured via a SOD assay kit (cat. #706003; Cayman Chemical Company, Ann Arbor, MI, USA) and analyzed using a microplate reader (BioTek Instruments, Inc., Winooski, VT, USA) at 450 nm. MDA levels were evaluated using an MDA assay kit (cat. #700870; Cayman Chemical Company, Ann Arbor, MI, USA) and analyzed at 530 nm. GPx activity was assessed spectrophotometrically by monitoring NADPH oxidation at 340 nm using the Glutathione Peroxidase Assay Kit (cat. #703102) from Cayman Chemicals (Ann Arbor, MI, USA). All analyses were conducted in triplicate across three different experimental protocols.

### 2.7. ELISA Assays

The expression of key proteins, including cytochrome c (#BMS263), Bcl-2 (#BMS244-3), Bid (#EH44RB), IL-6 (#EH2IL6), TNFα (#BMS607-3), VEGFα (#BMS277-2), and BNP (#EHNPPB), was assessed using Invitrogen ELISA (Thermo Fisher Scientific, Waltham, MA, USA). The hiPSC-vCMs (1.25 × 10^5^ cells) were plated in 24-well plates and cultured to 70–80% confluence. After 24 h of incubation, the cells were lysed. After lysing and centrifuging the cells, the supernatants were used for ELISA analysis. Protein expression levels were normalized to cell count to ensure accurate quantification. All assays were conducted in duplicate across three independent experiments.

### 2.8. Statistical Analysis

The data are presented as the mean ± standard error (SE). Statistical analyses were conducted utilizing Prism 10.1 software (GraphPad Software, San Diego, CA, USA). A statistical analysis was performed using one-way ANOVA to compare multiple experimental groups with independent samples that followed a normal distribution, as verified by the Shapiro–Wilk test. Tukey’s post hoc test was subsequently applied to identify significant differences among the groups and ensure precise pairwise comparisons. A quantitative PCR statistical analysis was performed using an unpaired Student’s *t*-test, with a significance threshold set at a *p*-value of ≤0.05. The statistical analyses were performed with a minimum required power level of 0.8, and all calculated power values exceeded 0.95 across the analyses. No blinding procedures were implemented during experimental procedures or data analysis. The objective nature of the outcome measures (MTT assays, qPCR, ELISA, and spectrophotometric enzyme activity measurements) minimized the potential for subjective interpretation bias.

## 3. Results

**Figure 1:**
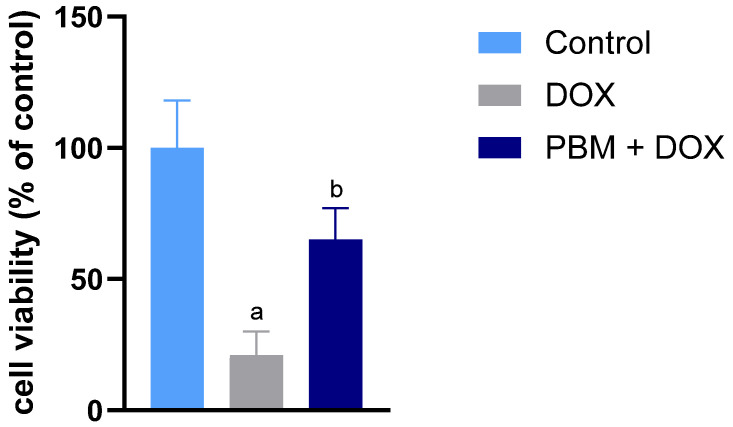
Effect of PBM on DOX-treated hiPSC-vCM viability using the MTT method. Data are presented as a percentage of the mean ± standard error (SE) of *n* = 3; ^a^ *p* < 0.05 vs. control; ^b^ *p* < 0.05 vs. DOX.

The impact of DOX on the viability of human-induced pluripotent stem cell-derived ventricular cardiomyocytes (hiPSC-vCMs), both in the presence and absence of PBM, was evaluated by an MTT assay. A significant reduction in cell viability was noted in the group treated solely with DOX. In contrast, the group receiving both PBM and DOX exhibited higher cell viability than those treated only with DOX (Figure 1). 

Table 1 encompasses all gene expression data from the distinct signaling pathways examined in this study. DOX exposure and PBM irradiation affected apoptosis signaling at the transcriptional and post-transcriptional levels. Significant increases in mRNA expression levels of *TP53* (*Tumor Protein P53*, a tumor suppressor gene linked to apoptosis) were observed in the DOX-treated cells compared to the control group. Preconditioning with PBM reduced *TP53* mRNA levels compared to the DOX cells, though these levels remained higher than in the control group. The administration of DOX also elevated pro-apoptotic *BAX* (*Bcl-2-associated X Protein*) mRNA expression compared to the control group. At the same time, PBM+DOX cells displayed a significant reduction in *BAX* mRNA levels compared to the other experimental groups. The expression of *FAS* (or *CD95*) mRNA, a pro-apoptotic receptor, was upregulated in the DOX cells but remained unchanged in the PBM+DOX cells compared to other experimental conditions.

**Figure 2:**
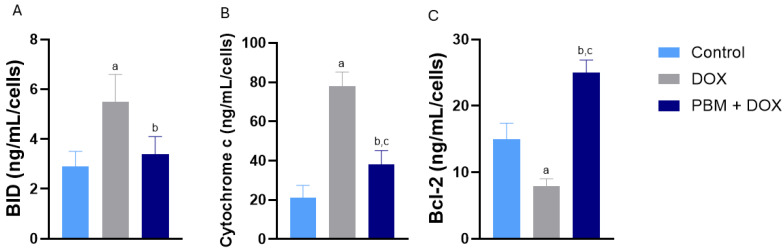
Effect of PBM on DOX-treated hiPSC-vCMs concerning pro-apoptotic BID (**A**) and cytochrome c (**B**), and anti-apoptotic Bcl-2 (**C**) proteins for all experimental groups. Protein expression was normalized to cell count. Data are presented as the mean ± standard error (SE) of *n* = 3; ^a^ *p* < 0.05 vs. control; ^b^ *p* < 0.05 vs. DOX; and ^c^ *p* < 0.05 vs. control.

Regarding protein expression, BID (BH3 Interacting Domain Death Agonist), another prominent pro-apoptotic protein of the Bcl-2 family, exhibited increased levels following DOX exposure but decreased when PBM was applied before DOX (Figure 2A). Cytochrome C protein expression, a component of the respiratory chain and activator of apoptosis, was higher in DOX-treated cells compared to other groups, with PBM preconditioning leading to a decrease in cytochrome C protein levels to control levels (Figure 2B). Conversely, BCL-2 protein expression (B-cell lymphoma 2, an anti-apoptotic factor) was significantly elevated in the PBM+DOX cells compared to other experimental groups (Figure 2C).

The exposure to DOX significantly elevated the mRNA levels of the pro-inflammatory cytokines *IL-6* and *TNF* compared to the control group (Table 1). Nonetheless, in the PBM+DOX cells, the expression levels of Interleukin-6 (IL-6) and Tumor Necrosis Factor (TNF) mRNA were reduced to comparable levels in the control group. Although the *Tumor Necrosis Factor Receptor Superfamily Member 1A (TNFRSF1A)* mRNA was downregulated in the DOX-treated cells relative to the controls, PBM preconditioning did not significantly affect mRNA expression of the TNF receptor (Table 1).

**Figure 3:**
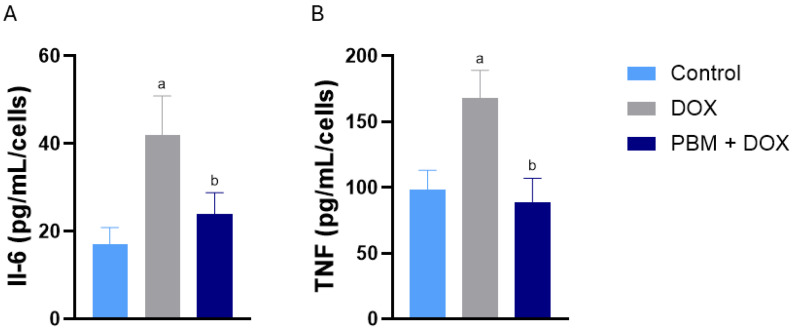
Effect of PBM on DOX-treated hiPSC-vCMs in proteins associated with the inflammatory response. ELISA data are provided for IL-6 (**A**) and TNF (**B**) across all experimental groups. Protein expression was normalized to cell count. Data are presented as mean ± standard error (SE) of *n* = 3; ^a^ *p* < 0.05 vs. control; ^b^ *p* < 0.05 vs. DOX.

Following DOX exposure, the protein levels of IL6 and TNF-alpha rose, which corresponded with their mRNA expression. The anti-inflammatory effects of PBM altered the expression of these cytokines, leading to a reduction in their protein levels after irradiation (Figure 3A and Figure 3B, respectively).

DOX exposure significantly inhibited the mRNA expression of the antioxidant enzymes *Glutathione Peroxidase 4 (GPX4), Catalase (CAT), and Superoxide dismutase 1 (SOD1)*. In the PBM+DOX group, however, the mRNA levels of *GPX4, CAT, and SOD1* were restored to levels comparable to or exceeding those observed in the control group (Table 1). While DOX administration elevated the levels of *Heat Shock Protein Family A (Hsp70) Member 1A/B (HSPA1A/B)* mRNA, a marker of oxidative stress, pretreatment with PBM significantly reduced *HSPA1A/B* mRNA levels to match those of the control group.

**Figure 4:**
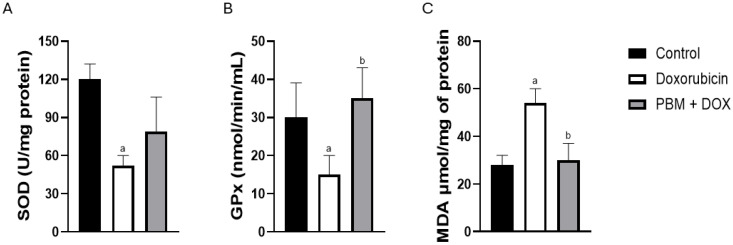
PBM affects proteins related to oxidative stress and antioxidant responses in DOX-treated hiPSC-vCMs. Measurements of antioxidant activities for SOD (**A**) and GPx (**B**) in hiPSC-vCMs treated with DOX or pre-conditioned with PBM+DOX. The MDA content (**C**) was assessed in all experimental groups. Data are presented as the mean ± standard error (SE) of *n* = 3; ^a^ *p* < 0.05 vs. control; ^b^ *p* < 0.05 vs. DOX.

Furthermore, the activity of antioxidant enzymes was evaluated across all experimental groups. After exposure to DOX, the activity of superoxide dismutase (SOD) decreased; however, PBM irradiation had no effect compared to the other experimental groups (Figure 4A). Moreover, glutathione peroxidase (GPx) activity decreased after DOX exposure, and a significant increase was observed following PBM preconditioning (Figure 4B). Interestingly, DOX treatment increased malondialdehyde (MDA) levels, while PBM significantly reduced MDA (Figure 4C). 

The analysis of metabolism-related genes revealed that exposure to DOX significantly reduced the mRNA levels of *Hexokinase 1 (HK1)* and *Phosphofructokinase (PFKM)*, key enzymes involved in glycolysis, compared to the control group (Table 1). Nevertheless, preconditioning with PBM only increased *PFKM* expression in the PBM+DOX cells. Furthermore, DOX strongly downregulated *Uncoupling Protein 2 (UCP-2)* mRNA; however, PBM+DOX cells exhibited a significantly higher increase in *UCP-2* expression than the DOX and control groups (Table 1). In contrast, neither DOX nor PBM significantly affected the mRNA expression of *Glyceraldehyde-3-Phosphate Dehydrogenase (GAPDH), NADH: Ubiquinone Oxidoreductase Subunit A3 (NDUFA3), Solute Carrier Family 2 Member 1 (SLC2A1),* or *Tafazzin (TAZ)*.

In the DOX-treated cells, *VEGFa (Vascular Endothelial Growth Factor A)* mRNA expression did not change, while an increase was noted in the PBM+DOX cells (Table 1). VEGF protein levels increased in both DOX-treated groups, but without statistical significance. Exposure to DOX resulted in a significant reduction in the mRNA expression of *ATP2A2* (*ATPase Sarcoplasmic/Endoplasmic Reticulum Ca2+ Transporting 2*, also known as SERCA2), a gene essential for calcium reuptake into the sarcoplasmic reticulum, compared to the control group (Table 1). In contrast, preconditioning with PBM before DOX administration effectively restored *ATP2A2* mRNA expression to levels similar to those in the control group. In contrast, DOX treatment downregulated the mRNA levels of *RYR-2* (*Ryanodine Receptor 2 gene*), a calcium release channel, and *SLC8A1* (*Solute Carrier Family 8 Member 1 gene*), a sodium-calcium exchanger; however, PBM preconditioning did not result in any significant modifications to their expression levels relative to the controls (Table 1). Furthermore, no alterations were detected in the mRNA levels of *CASQ2* (*Calsequestrin 2*, involved in calcium storage) or *PLN* (*Phospholamban*, a calcium pump regulator) across all experimental groups.

The administration of DOX led to significant changes in the expression levels of genes involved in regulating the extracellular matrix (ECM). The DOX cells exhibited increased mRNA levels for *Matrix Metalloproteinase 9 (MMP-9), Tenascin C (TNC), Transforming Growth Factor Beta 1 (TGFB1),* and *Collagen Type III Alpha 1 Chain (COL3A1)* (Table 1). Conversely, the mRNA expression of *Collagen Type I Alpha 1 Chain (COL1A1)* was diminished in the DOX-treated cells compared to the control group. Preconditioning with PBM has been observed to modulate specific alterations. PBM+DOX cells exhibited greater mRNA expression of *MMP9* compared to the other experimental groups; however, their *TNC* levels were lower, resembling those of the control group (Table 1). The mRNA levels of *TGFB1* and *COL1A1* remained unchanged in PBM+DOX cells.

The mRNA expression levels of most genes associated with cardiac hypertrophy were significantly regulated when compared to cells treated with DOX and those in the control group. The DOX-treated cells exhibited reduced mRNA levels for *Angiotensin-converting enzyme (ACE), Cardiac hypertrophy protein 2 (CHP2), Insulin-like growth factor 1 (IGF-1), Myosin heavy chain 6 (MYH6),* and *Endothelin (EDN1)* compared to the control group (Table 1). *MYH6* and *EDN1* mRNA expressions were significantly elevated in the PBM+DOX cells compared to the DOX-treated cells. Conversely, the expression levels of mRNA for *Angiotensin-converting enzyme 2 (ACE2), Myosin heavy chain 7 (MYH7),* and *Angiotensin II receptor type 1 (AGTR1A)* were increased in the DOX group when compared to the control cells (Table 1).

**Figure 5:**
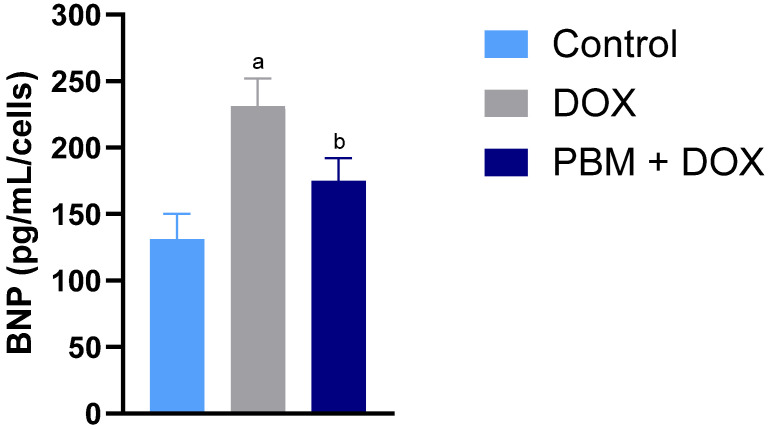
ELISA used to assess the effect of PBM on BNP levels in all hiPSC-vCM groups. Results are displayed as mean ± SE of *n* = 3; ^a^ *p* < 0.05 vs. control; ^b^ *p* < 0.05 vs. DOX.

Cells exposed to DOX exhibited higher mRNA levels of several genes associated with the hypertrophic state in cardiomyocytes, including Calcineurin Binding Protein 1 (CABIN1) and Natriuretic peptide B (NPPB) (Table 1), compared to the control group. The *NFATC3* (*Nuclear Factor of Activated T-Cells 3 gene*) mRNA levels increased only in PBM+DOX cells (Table 1). Exposure to DOX resulted in a decrease in *Natriuretic Peptide A (NPPA)* expression. Notably, following DOX treatment, PBM decreased the mRNA levels of the natriuretic peptide genes *NPPB* and *NPPA* (Table 1) after exposure to DOX. Corroborating *NPPB* expression, BNP levels significantly decreased in the PBM+DOX cells compared to DOX-exposed cells (Figure 5). 

## 4. Discussion

This study investigated the impact of photobiomodulation preconditioning on hiPSC-derived ventricular cardiomyocytes exposed to doxorubicin. These cardiomyocytes mimic many physiological and pathological features of natural cardiomyocytes, though they are not fully mature [19]. Currently, advances in human organoids—spheroids produced through cardiac differentiation from self-assembled human pluripotent stem cell (hPSC) aggregates or spherical microtissues formed by aggregating pre-differentiated cardiac cells—offer significant potential [20,21,22,23]. Nonetheless, this three-dimensional (3D) culture model has not yet achieved widespread accessibility. The rationale behind subjecting hiPSC-vCMs to acute DOX exposure is twofold. Firstly, acute cardiotoxicity may serve as an early indicator of the complex processes that eventually lead to chronic myocardial dysfunction. Secondly, identifying potential cardioprotective strategies at this initial stage could be clinically significant, especially when combined with preconditioning interventions. 

PBM has demonstrated significant promise as a cost-effective and straightforward method for mitigating DOX-induced cardiotoxicity. The PBM’s effectiveness appears to be related to its ability to initially regulate gene expression associated with heart dysfunction, thereby enhancing cell survival. While DOX treatment altered the expression of 32 genes (80%), primarily by increasing those related to apoptosis and inflammation, PBM influenced 20 genes (50%), notably reducing stress markers such as *HSP1A1, TNC,* and *TP53*. Notably, PBM significantly reduced the expression of natriuretic peptides, suggesting its potential role in alleviating hypertrophic stress and enabling cytoprotection in cardiomyocytes affected by DOX.

A significant finding of this study was the enhanced cell viability observed with PBM preconditioning before DOX exposure. The precise mechanisms by which cell death impacts DOX-CM remain inadequately understood. While necrosis likely contributes to the cell death observed in DOX-treated cardiomyocytes [24], apoptosis remains the most well-characterized type of cell death in DOX cardiotoxicity [25]. Our data suggest that PBM reduces apoptotic signaling by lowering the expression of pro-apoptotic genes, such as *BAX* and *TP53*, as reported by others [26,27], while increasing the expression of anti-apoptotic factors, including Bcl-2. Moreover, during apoptosis, the balance between pro-apoptotic factors (such as Bax and Bid) and the anti-apoptotic Bcl-2 is essential for activating apoptosis pathways, which can result in the release of cytochrome c from the mitochondria, a crucial step in the apoptosis pathway [28]. Additionally, PBM decreased Bid levels, indicating its potential protective role. While other mechanisms of cell death, such as autophagy, ferroptosis, and mitophagy, may contribute to DOX-induced cardiotoxicity, further research is necessary to understand the effects of PBM on cell death signaling pathways [29,30].

DOX treatment negatively impacted oxidative stress responses by altering the expression of several antioxidant enzymes [31,32]. PBM may counteract these effects by reducing oxidative markers and enhancing the expression and activity of antioxidant genes, indicating its potential to alleviate DOX-induced oxidative damage [33,34]. Notably, key markers, such as malondialdehyde (MDA), were significantly reduced with PBM treatment, further substantiating these observations. DOX also increased the expression of *HSPA1A/B,* which encodes the protective protein HSP70, and this effect was diminished by PBM [35]. This evidence suggests that PBM may mitigate cellular stress induced by DOX by reducing *HSPA1A/B* mRNA levels, a marker of oxidative stress. Furthermore, our findings showing increased mRNA levels of antioxidant enzymes and higher GPx activity, and other published data [36,37] suggest that PBM may have potential cardioprotective effects against ROS produced by DOX.

Calcium handling, critical for cardiac function, is disrupted by DOX, which can lead to contractile disturbances and cardiac hypertrophy [38,39]. PBM appears to oppose these changes, as indicated by its modulation of the sarcomeric *MYH6* gene expression, which is linked to the development of cardiomyopathy [40]. DOX also upregulated hypertension-associated genes in the renin–angiotensin system and endothelin. Although PBM did not alter RAS component gene expression, it decreased *EDN1* expression, which may contribute to improved endothelial function after DOX in vivo treatment [41].

Our data analysis indicated that DOX significantly increased the mRNA expression levels of the genes *IGF1, MYH7, NPPA*, and *NPPB*, corroborating findings from previous studies. These genes are associated with cellular growth, development, and cardiac function. Notably, the *NPPA* and *NPPB* genes encode atrial natriuretic peptide (ANP) and brain natriuretic peptide (BNP), respectively, which are biomarkers associated with cardiac complications in patients treated with DOX [29,42]. The observed increase in mRNA levels of these peptides aligns with clinical indicators of cardiac stress and damage. Importantly, our data show that PBM reduced the expression of natriuretic peptides’ mRNA. Elevated BNP levels are clinically used to diagnose myocardial damage and heart failure, serving as early markers of cardiac issues [43]. Importantly, PBM pretreatment followed by DOX exposure resulted in decreased BNP protein levels. These findings are relevant to readers because in vivo data show that a 46% decrease in BNP levels is associated with a better prognosis in patients with acute decompensated heart failure [44]. This evidence indicates that PBM may effectively reduce DOX-related cardiotoxicity.

Considering the well-documented anti-inflammatory effects of PBM in contrast to the pro-inflammatory impact of DOX, our findings demonstrate that PBM can mitigate inflammation by reducing the mRNA and protein levels of the pro-inflammatory cytokines IL-6 and TNF, as widely reported [45,46,47]. This data highlights PBM’s potential to counteract the inflammatory response induced by DOX in hiPSC-CMs. Furthermore, photobiomodulated extracellular matrix (ECM) genes were related to remodeling, decreasing *TNC* while promoting *COL3A1* expression. Tenascin can modulate cellular signaling by inducing pro-inflammatory cytokines and is promptly upregulated in response to tissue injury [48]. The observed reduction in *TNC* mRNA expression, concurrent with decreased cytokine levels, suggests that PBM may have therapeutic potential for alleviating DOX-induced cardiac inflammation.

DOX-induced cardiotoxicity leads to changes in metabolism-related transcripts [49]. Cells exposed to DOX show reduced expression of the *HK1* and *PFKM* genes, which encode enzymes essential for glycolysis, potentially impairing energy production and apoptosis protection. Initially, *GAPDH* was considered an alternative housekeeping gene (HKG) for the PCR protocol. Despite its consistent expression across experimental groups, we presented it solely as a gene involved in the metabolic pathway due to its high standard deviation and coefficient of variation, different from those of *18S* rRNA. Previous studies have reported varying expression of the *GAPDH* gene with or without PBM [50,51]. DOX may also inhibit components of the electron transport chain. PBM showed promising results in enhancing mitochondrial energy metabolism, as indicated by the increased expression of *UCP-2*. These findings align with previous research suggesting that PBM can improve cellular energy dynamics [52,53]. 

While this study offers significant insights into the cardioprotective effects of PBM in mitigating DOX-induced cardiotoxicity, several limitations must be carefully considered. Firstly, the experimental setup involved a single-dose administration of DOX, which may not fully encompass the diverse range of cardiotoxic effects seen in clinical settings, including variations in dosage and treatment duration. This constraint limits the ability to generalize the findings across more complex clinical scenarios. Secondly, although hiPSC-vCMs serve as a valuable model for investigating cardiac responses, they lack the complete physiological complexity and molecular signaling pathways found in mature cardiomyocytes or in vivo cardiac tissue. Employing 3D culture models, such as hPSC-derived cardiac spheroids [54,55], could enhance the translational relevance of future research. Another significant limitation is the selection and validation of housekeeping genes (HKGs) for quantitative PCR (qPCR) analysis. While *18S* rRNA proved adequate for this study, additional validation of HKG would bolster the accuracy and reliability of gene expression data. Furthermore, more in-depth protein quantifications and assays, such as TUNEL or caspase activity measurements, would provide more robust evidence to support the cellular mechanisms affected by PBM. Despite these limitations, this study highlights the potential of preconditioning PBM as a cost-effective and non-invasive approach to mitigating DOX-induced cardiotoxicity. Addressing these limitations in future research will be crucial for translating these findings into effective clinical applications.

## 5. Conclusions

This study underscores photobiomodulation (PBM) preconditioning as a promising, non-invasive strategy for mitigating doxorubicin (DOX)-induced cardiotoxicity by enhancing cell viability, reducing oxidative stress, and suppressing pro-inflammatory and pro-apoptotic markers in hiPSC-derived ventricular cardiomyocytes.

## Figures and Tables

**Table 1:** Quantitative results of the PCR *TaqMan* assays conducted across multiple experimental groups, highlighting changes in gene expression in response to DOX treatment and PBM preconditioning. Data are expressed as fold changes relative to the control group (set as 1). The values are shown as the mean ± standard error (SE) of *n* = 3.

Signaling Pathway	*Gene*	DOX (Mean ± SE)	PBM+DOX (Mean ± SE)
**Angiogenesis**	*VEGFA*	0.96 ± 0.12	1.67 ± 018 ^b,c^
**Apoptosis**	*BAX*	1.97 ± 0.28 ^a^	0.67 ± 0.06 ^b,c^
	*FAS*	1.72 ± 0.33 ^a^	1.38 ± 0.51
	*TP53*	3.34 ± 0.48 ^a^	1.76 ± 0.35 ^b,c^
**Calcium kinetics**	*ATP2A2*	0.47 ± 0.11 ^a^	0.94 ± 0.08 ^b^
	*CASQ2*	0.90 ± 0.12	0.76 ± 0.23
	*PLN*	1.12 ± 0.17	1.01 ± 0.28
	*RYR-2*	0.49 ± 0.17 ^a^	0.84 ± 0.21
	*SLC8A1*	0.63 ± 0.10 ^a^	0.56 ± 0.35
**Oxidative stress**	*CAT*	0.24 ± 0.04 ^a^	1.64 ± 0.23 ^b,c^
	*GPX4*	0.60 ± 0.11 ^a^	1.89 ± 0.23 ^b,c^
	*HSPA1A/B*	2.19 ± 0.56 ^a^	1.21 ± 0.15 ^b^
	*SOD1*	0.39 ± 0.09 ^a^	0.89 ± 0.12 ^b^
**Cardiac hypertrophy**	*ACE*	0.43 ± 0.22 ^a^	0.93 ± 0.36
	*ACE2*	1.94 ± 0.30 ^a^	1.56 ± 0.41
	*AGTR1A*	1.80 ± 0.42 ^a^	1.55 ± 0.42
	*CABIN1*	1.67 ± 0.21 ^a^	1.42 ± 0.64
	*CHP2*	0.52 ± 0.16 ^a^	0.79 ± 0.42
	*EDN1*	0.65 ± 0.15 ^a^	1.36 ± 0.17 ^b^
	*IGF1*	0.44 ± 0.07 ^a^	0.79 ± 0.26
	*MYH6*	0.65 ± 0.11 ^a^	1.73 ± 0.30 ^b^
	*MYH7*	1.69 ± 0.19 ^a^	1.24 ± 0.37
	*NFATC3*	1.24 ± 0.14	1.82 ± 0.21 ^b,c^
	*NPPA*	2.76 ± 0.38 ^a^	1.85 ± 0.27 ^b^
	*NPPB*	2.98 ± 0.70 ^a^	1.74 ± 0.25 ^b^
**Inflammation**	*IL-6*	3.14 ± 0.26 ^a^	1.34 ± 042 ^b^
	*TNFRSF1a*	0.67 ± 0.17 ^a^	0.82 ± 0.25
	*TNF*	2.36 ± 0.24 ^a^	1.41 ± 0.21 ^b^
**Extracellular matrix**	*COL1A1*	0.46 ± 0.12 ^a^	0.86 ± 0.22
	*COL3A1*	1.35 ± 0.11 ^a^	1.87 ± 0.23 ^b,c^
	*MMP9*	1.35 ± 0.14 ^a^	1.77 ± 0.14 ^b,c^
	*TGFB1*	1.41 ± 018 ^a^	1.13 ± 0.12
	*TNC*	2.84 ± 0.48 ^a^	1.54 ± 0.35 ^b^
**Cell metabolism**	*GAPDH*	0.89 ± 0.31	0.69 ± 0.44
	*HK1*	0.45 ± 0.22 ^a^	0.98 ± 0.31
	*NDUFA3*	0.77 ± 0.25	0.83 ± 0.36
	*PFKM*	0.43 ± 0.27 ^a^	1.13 ± 0.21 ^b^
	*SLC2A1*	1.56 ± 0.57	1.41 ± 0.49
	*TAZ*	1.24 ± 0.31	0.97 ± 0.34
	*UCP-2*	0.33 ± 0.02 ^a^	1.94 ± 0.36 ^b,c^

^a^ *p* < 0.05 vs. control; ^b^ *p* < 0.05 vs. DOX; and ^c^ *p* < 0.05 vs. control.

## Data Availability

The data supporting the findings of this study are available in this article. All other relevant source data can be obtained from the corresponding author upon request.

## Abbreviations

The following abbreviations are used in this manuscript:

DOX	Doxorubicin
PBM	Photobiomodulation
mRNA	Messenger ribonucleic acid
hiPSC-vCMs	Human-induced pluripotent stem cell-derived ventricular cardiomyocytes
